# Correction: Franceschi et al. Sedoheptulose Kinase SHPK Expression in Glioblastoma: Emerging Role of the Nonoxidative Pentose Phosphate Pathway in Tumor Proliferation. *Int. J. Mol. Sci.* 2022, *23*, 5978

**DOI:** 10.3390/ijms26031044

**Published:** 2025-01-26

**Authors:** Sara Franceschi, Francesca Lessi, Mariangela Morelli, Michele Menicagli, Francesco Pasqualetti, Paolo Aretini, Chiara Maria Mazzanti

**Affiliations:** 1 Fondazione Pisana per la Scienza, 56017 Pisa, Italy; f.lessi@fpscience.it (F.L.); m.morelli@fpscience.it (M.M.); m.menicagli@fpscience.it (M.M.); p.aretini@fpscience.it (P.A.); c.mazzanti@fpscience.it (C.M.M.); 2 Department of Radiation Oncology, Azienda Ospedaliera Universitaria Pisana, University of Pisa, 56126 Pisa, Italy; f.pasqualetti@ao-pisa.toscana.it; 3 Department of Oncology, University of Oxford, Oxford OX3 7DQ, UK

In the original publication [1], there was a mistake in Figure 3B as published. In the vector row, panels T98G and U118 appeared to show the same specimen, although with a different magnification factor. The corrected Figure 3B appears below. The authors state that the scientific conclusions are unaffected. This correction was approved by the Academic Editor. The original publication has also been updated.


